# A resource of induced pluripotent stem cell (iPSC) lines including clinical, genomic, and cellular data from genetically isolated families with mood and psychotic disorders

**DOI:** 10.1038/s41398-023-02641-w

**Published:** 2023-12-16

**Authors:** Sevilla D. Detera-Wadleigh, Layla Kassem, Emily Besancon, Fabiana Lopes, Nirmala Akula, Heejong Sung, Meghan Blattner, Laura Sheridan, Ley Nadine Lacbawan, Joshua Garcia, Francis Gordovez, Katherine Hosey, Cassandra Donner, Claudio Salvini, Thomas Schulze, David T. W. Chen, Bryce England, Joanna Cross, Xueying Jiang, Winston Corona, Jill Russ, Barbara Mallon, Amalia Dutra, Evgenia Pak, Joe Steiner, Nasir Malik, Theresa de Guzman, Natia Horato, Mariana B. Mallmann, Victoria Mendes, Amanda L. Dűck, Antonio E. Nardi, Francis J. McMahon

**Affiliations:** 1 Genetic Basis of Mood & Anxiety Disorders Section, Human Genetics Branch, National Institute of Mental Health Intramural Research Program, National Institutes of Health, 35 Convent Drive, Bethesda, MD 20892 USA; 2 Laboratorio de Panico e Respiracao, Instituto de Psiquiatria, Universidade Federal do Rio de Janeiro, Rio de Janeiro, 22410-003 Brazil; 3 Department of Psychiatry and Human Behavior, Alpert Medical School of Brown University, Providence, RI 02903 USA; 4 Institute of Psychiatric Phenomics and Genomics, LMU Munich, 80336 München, Germany; 5 Department of Psychiatry and Behavioral Sciences, Upstate University Hospital, Syracuse, NY 13210 USA; 6 Department of Psychiatry and Behavioral Sciences, Johns Hopkins University, Baltimore, MD 21287 USA; 7 Center for Scientific Review, Neurotechnology and Vision Branch, National Institutes of Health, Bethesda, MD 20892 USA; 8 Cytogenetics and Microscopy Core, National Human Genome Research Institute, National Institutes of Health, Bethesda, MD 20892 USA; 9 Neurotherapeutics Development Unit, NINDS, National Institutes of Health, Bethesda, MD 20892 USA

**Keywords:** Stem cells, Genetics, Neuroscience

## Abstract

Genome-wide (GWAS) and copy number variant (CNV) association studies have reproducibly identified numerous risk alleles associated with bipolar disorder (BD), major depressive disorder (MDD), and schizophrenia (SCZ), but biological characterization of these alleles lags gene discovery, owing to the inaccessibility of live human brain cells and inadequate animal models for human psychiatric conditions. Human-derived induced pluripotent stem cells (iPSCs) provide a renewable cellular reagent that can be differentiated into living, disease-relevant cells and 3D brain organoids carrying the full complement of genetic variants present in the donor germline. Experimental studies of iPSC-derived cells allow functional characterization of risk alleles, establishment of causal relationships between genes and neurobiology, and screening for novel therapeutics. Here we report the creation and availability of an iPSC resource comprising clinical, genomic, and cellular data obtained from genetically isolated families with BD and related conditions. Results from the first 324 study participants, 61 of whom have validated pluripotent clones, show enrichment of rare single nucleotide variants and CNVs overlapping many known risk genes and pathogenic CNVs. This growing iPSC resource is available to scientists pursuing functional genomic studies of BD and related conditions.

## Introduction

GWAS have shown that common alleles contribute to the polygenic architecture of complex psychiatric illnesses such as BD [1, 2], MDD [3], and SCZ [4, 5]. Although each individual allele confers a small risk, together they account for 5-30% of the phenotypic variance [6, 7]. Increasing sample size should increase the proportion of phenotypic variance explained by common alleles [8], but most projections fall far short of heritability estimates from twin and family studies [9].

Some of this missing heritability may reflect rare, higher-risk variants largely missed in GWAS [9]. These include single nucleotide variants (SNVs), small insertions/deletions (indels), and CNVs. Various CNVs, such as those on 1q21, 15q11.2, 16p11.2, and 22q11.2, have been shown to substantially increase the risk for neurodevelopmental and neuropsychiatric disorders [7, 10–15]. In addition, rare SNVs and indels have been revealed in ASD, BD and SCZ [16–19].

However, the contribution of rare variants to heritability of neuropsychiatric disorders has not been fully resolved, due in part to the massive sample sizes typically required to identify rare disease variants. Detection of low-frequency deleterious alleles is theoretically possible in smaller samples when allele frequencies are increased by genetic drift [20]. The validity of this theory is supported by several studies in genetically isolated populations such as the Finns [21], Ashkenazim [22], and Old Order Amish [23].

Despite the substantial progress in the identification of risk alleles, little is known about their neurobiological impacts in the brain. The Enhancing Neuroimaging Genetics through Meta-Analysis CNVs (ENIGMA-CNV) working group has recently reported CNV-associated features in brain structure [24]. Multiple studies have analyzed transcriptome changes in patients’ postmortem brains, providing rich data on perturbed gene networks in BD, SCZ and other psychiatric disorders [25–29] and establishing statistical connections with some risk alleles. These findings, however, are derived from only one time point in the illness and are confounded by various factors, including the loss of viable cells which precludes experimental manipulations and capture of dynamic biological mechanisms. Additionally, embedded within the complex milieu of an individual’s life are medication history and cause of death. Cross-sectional studies cannot differentiate between structural changes that cause disease and those that are a consequence of the disease or its treatment.

The use of human-derived iPSC (hiPSC) permits a complementary approach and may recapitulate certain features of neural cells in neuropsychiatric disorders. Pluripotency ensures a renewable cellular reagent that can be differentiated into living, disease-relevant cells that carry the full complement of the donor’s germline genetic variation. iPSCs and their cellular derivatives can be extensively phenotyped and studied experimentally either in a monolayer or 3D format, enabling the establishment of causal relationships between risk alleles and cellular neurobiology. Several recent studies of iPSC-derived neurons carrying known neuropsychiatric CNVs [30, 31] or other risk alleles [32–34] demonstrate the promise of this approach, but there is an urgent need to characterize a broader range of risk alleles, particularly those high-impact, functionally damaging variants, and identify convergent neurobiological effects potentially amenable to therapeutic remediation.

In 2009 we initiated a project to establish a database of clinically phenotyped and genetically characterized families from Amish and Mennonite population isolates, ascertained through probands with BD or related illnesses.

In addition to strong founder effects that increase frequencies of some deleterious alleles [35] these populations offer special advantages for the study of psychiatric disorders: i) minimal or no confounding effects on psychiatric diagnosis by substance abuse; ii) families live in well-circumscribed agrarian societies with relatively uniform socio-economic circumstances, and in-marriage from the outside population is rare which reduces genetic heterogeneity; and, iii) families are large which facilitates analysis of genetic transmission of disease.

The present study has three main goals: a) to ascertain, clinically assess, and genetically profile BD and related conditions in the Amish and Mennonite population isolates, b) to identify rare, high-risk variants, and c) to develop an iPSC resource that comprises a “living catalog” of risk alleles providing a renewable cellular platform for systematic studies of the molecular and neurobiological effects of risk variants.

Here, we present a sample resource which includes a clinical database of psychiatric, medical, and neuropsychological data, and a catalog of rare genetic variants identified by exome sequencing and SNP array analysis on probands and their extended families. This resource includes a biobank of iPSC clones that provides a sustainable platform for in vitro modeling studies and screening for improved therapeutics in human-derived cells. These data and biomaterials are available to scientists pursuing functional genomic studies of BD and related neuropsychiatric disorders.

## Materials and methods

### Amish-Mennonite Bipolar Genetics (AMBiGen) Project

In 2009, the Human Genetics Branch of the National Institute of Mental Health-Intramural Research Program (NIMH-IRP) established the Amish-Mennonite Bipolar Genetics (AMBiGen) Project to recruit families afflicted with BD and related neuropsychiatric disorders for genetic studies. BD is a common, complex, disabling disease marked by cycles of mania and depression and varied ages of onset, symptom severity, episode frequencies and responses to therapy [36]. Twin and adoption studies in BD have shown over 75% heritability [37, 38], ~30% of which is explained by common SNPs [1].

Ascertainment is directed towards genetically isolated Anabaptist communities in the Americas that represent mostly Amish and Mennonites, but also include other Anabaptist groups who trace their ancestry to Western Europe. The collection includes 62 individuals from the Pennsylvania (PA) Lancaster County Old Order Amish, that was recently subjected to a genome-wide association study for mood disorders [39], Amish living in Ohio, Indiana, other parts of PA, and other regions of the US, and Mennonites living in the US, Canada, and Brazil. In 2015, the Mennonite population in the US and Brazil has grown to 539,000 and 15,000, respectively. The genetic relationships among our study participants have been evaluated previously [35].

#### Ascertainment and recruitment

All participants are studied under a protocol approved by the NIH Institutional Review Board (80-M-0083). Study volunteers are recruited through advertisements, mental health treatment providers, and residential care facilities that focus on treatment of Anabaptists. Ancestry and family relationships are provisionally assigned based on participants’ self-reports but are later confirmed genealogically [Anabaptist Genealogy Database (AGDB) [40] and Swiss Anabaptist Genealogical Association (SAGA) [41]] and molecularly by population principal components and allele-sharing analyses [42]. We employ a sequential ascertainment strategy beginning with an affected individual and extending to all available first-degree relatives. Additional family branches are ascertained based on relatives’ reports of potential additional cases. This leads to a sample enriched for BD and related illnesses, with many affected and unaffected relatives. We expect that these relatives would share many common risk alleles with the proband, but would segregate rare alleles in Mendelian proportions, thus enhancing the power to detect rare, high-risk alleles [43]. Further details of the ascertainment methods, including prescreening, enrollment, and informed consent are described under Supplementary Information.

### Clinical assessment and phenotyping

#### Clinical overview

Probands and putatively affected relatives are interviewed with the Diagnostic Interview for Genetics Studies (DIGS), a semi-structured instrument with high reliability for bipolar I (BPI), bipolar II (BPII), MDD and SCZ [44]. The Family Interview for Genetic Studies (FIGS) (https://www.nimhgenetics.org/interviews/figs/) is typically performed with a family informant to provide additional perspectives on affected relatives. These data, along with any available medical/psychiatric records, are reviewed independently by two clinicians who assign psychiatric diagnoses in a Best Estimate procedure [45]. In our experience, both reviewers agree 93% of the time on a diagnosis of a major mood or psychotic disorder. When they disagree, a third reviewer assigns the final diagnosis based on all available information.

Some families undergo additional clinical assessments. These include dimensional measures of psychopathology [Symptom Checklist 90 – Revised (SCL-90-R), Mood Disorder Questionnaire, and Past History Schedule]. If dimensional measures are suggestive of a previously unidentified mood disorder, a follow-up DIGS is completed when possible. Neurocognitive measures were selected to assess several domains and to be insensitive to differences in educational attainment and language typical of Anabaptist communities. Measures include seven tasks that assess executive functioning, spatial reasoning, verbal memory, reaction time, face memory, and face emotion recognition: DANVA [46], Flanker [47], Penn Face Memory Test [48], Trails Making Test-Part A (TMT-A) [49], California Verbal Learning Test (CVLT) [50], and WASI-II Matrix Reasoning [51]. Before administering the neurocognitive battery, euthymia is assessed using the Beck Depression Inventory-II [52] and the Young Mania Questionnaire [53]. All available assessments are provided to Best Estimate reviewers.

#### Exome sequencing and QC pipeline

Genomic DNA (gDNA) was extracted from blood (or rarely, saliva) samples of consenting study participants using Gentra Puregene kit (Qiagen, MD). DNA concentration was measured in a NanoDrop spectrophotometer or by fluorescence using Qubit (Thermo Fisher Scientific, MA). gDNA from study participants were sent to our collaborator, Regeneron Genetics Center LLC (Tarrytown, NY), for exome sequencing and SNP genotyping. Due to poor DNA quality, or sex discrepancy, or contamination, 55 samples were excluded from the analysis. Exons in the remaining samples were captured using the IDT xGen Exome Research Panel v1.0 (Integrated DNA Technologies, Coralville, IA) and sequenced at >30X coverage on the Illumina HiSeq2500 platform (Illumina, San Diego, CA). Raw reads were mapped to GRCh38 using Burrows-Wheeler Alignment Tool (BWA) [54] and variants were called using the Genome Annotation Toolkit (GATK) Best Practices pipeline (https://software.broadinstitute.org/gatk/best-practices/). GATK’s Variant Quality Score Recalibration (VQSR) procedure was performed to extract superior quality variants. All Mendelian errors, genotypes with GQ < 20, DP < 10 and AB < 0.25/ > 0.75, and variants with >2% missing calls were excluded.

#### Rare variant, allele frequency and allelic enrichment in AMBiGen

Following quality control, variants were mapped to their cognate gene(s) and functionally annotated using Ensembl Variant Effect Predictor v104.3 (VEP) [55]. Functional variants (nonsynonymous, missense, frameshift, stop-gain, stop-loss) with maximum minor allele frequency (MAF) of <1% in any control dataset were classified as “rare variants” and included in this analysis. Rare variant analysis was directed solely toward genes located within significant BD and SCZ GWAS regions. Since genotyped samples represent related individuals and the focus here is on inherited risk, singleton variants were excluded.

Reference allele frequencies were drawn from the Anabaptist Variant Server (AVS) (edn.som.umaryland.edu/Anabaptist) (see Acknowledgment) and Genome Aggregation Database (gnomAD) based on unrelated non-Finnish Europeans [55] v2.1.1 which includes >55,000 sequenced exomes [The genome Aggregation Database (gnomAD) | MacArthur Lab]. Variants not found in either reference sample were classified as “private”.

AVS represents separate sample collections consisting mainly of Amish and Mennonites, totaling >10,000 individuals, from the following sources: University of Maryland (*n* = 7278); Clinic for Special Children (CSC), Lancaster County, PA (*n* = 930); Developmental Disorder Clinic (DDC), Geauga County, OH (*n* = 1426); Kansas Mennonites (*n* = 182, kindly provided by Michael H. Crawford) and the NIMH - AMBiGen sample (*n* = 997). NIMH samples were excluded from AVS allele-frequency data.

Variant enrichment ratio was measured by taking the AMBiGen MAF, adjusted for relatedness (ROADTRIPS version 1.2) [56, 57] divided by the MAF in either AVS or gnomAD v2.1.1 for unrelated non-Finnish Europeans [55].

#### Potential function of rare variant carrying genes and variant deleteriousness

To determine whether genes located in GWAS regions that carry rare variants have been shown to be dysregulated in BD and/or SCZ postmortem brains, we examined reported data from transcription-wide association studies (TWAS) and summary-based Mendelian randomization (SMR) analysis [2, 26, 58] and results are shown in Table 2 and Supplementary Table 1. In addition, we searched the TWAS Atlas that contains 22,247 genes, 257 traits, and >400,000 TWAS associations [59] [https://ngdc.cncb.ac.cn/twas/] (Table 2, Supplementary Table 1).

The potential deleteriousness of rare variants was ascertained by calculating the Combined Annotation-Dependent Depletion (CADD) (cadd.gs.washington.edu)-PHRED scores [60, 61] (Table 2, Supplementary Table 1).

#### SNP microarray genotyping and CNV analysis

Genotyping was done on the Illumina OmniExpress or GSA Human SNP arrays (Illumina, CA). PennCNV software [62] was used for CNV calling, with standard parameter settings. Samples with more than 10 large CNVs were excluded from this analysis as they might be due to technical problems. For the remaining samples we tested if any of our called CNVs overlapped with known pathogenic CNVs previously associated with neuropsychiatric disorders [14]. Since CNVs called from SNP arrays have imprecise breakpoints, if multiple smaller CNVs with the same copy number in the same sample overlapped the same known psychiatric CNV, these were merged into a single CNV before estimating total overlap.

#### Polygenic risk score (PRS)

PRS in AMBiGen was calculated by using the latest Psychiatric Genomics Consortium for Bipolar Disorder (PGC BIP) GWAS test statistics [2] based on European ancestry (https://pgc.unc.edu/for-researchers/download-results/). The PGC BIP test statistics and AMBiGen SNP array data were merged with variants on hg38 genomic positions yielding a total of 383,711 variants. Then summary statistics were clumped and PRS was calculated by PLINK v1.90b3.36 [63] with 10 different p-value thresholds.

To find the best-fit PRS, a logistic mixed model in GMMAT [64] package in R was used. The logistic mixed model of PRS for AMBiGen phenotypes yielded the most significant p-value and the greatest effect size when the PGC BIP *p*-value threshold of < 0.1 was used, under a broad affection status (BP-I, BP-II with single/recurrent depression, schizoaffective manic/bipolar/depressed, SCZ, BP-NOS, MDD recurrent).

#### Tissue specimens reprogrammed into iPSCs

The proband and at least one unaffected relative per family were requested to donate a tissue specimen for iPSC generation. From 2012-2019, this tissue was collected by dermal biopsy, cultured to produce fibroblast cell lines, then reprogrammed into iPSCs. Since 2019, iPSCs have been generated primarily from peripheral blood mononuclear cells (PBMCs) isolated from specimens collected in BD Vacutainer CPT tubes (BD Biosciences, CA). PBMCs were resuspended in CryoStor CS10 freezing medium (StemCell Technologies, Vancouver, Canada) in barcoded cryovials and stored in liquid nitrogen. Blood samples were also sent to Rutgers Cell and DNA Repository (RUDCR, NJ) for derivation of lymphoblastoid cell lines (LCLs) and banking of genomic DNA (gDNA), lymphocytes, and LCLs for distribution to qualified scientists.

#### Reprogramming of somatic cells into iPSCs, characterization of iPSCs, and development of web portal

Reprogramming of fibroblasts or PBMCs into iPSCs has been conducted mostly by the National Heart Lung and Blood Institute (NHLBI-NIH) iPSC Core using the CytoTune-iPS 2.0 Sendai Reprogramming kit (Thermo Fisher Scientific, MA).

iPSCs are characterized by examining the following factors: a) growth properties, b) sterility, c) absence of mycoplasma contamination, d) karyotype by either Giemsa staining (WiCell, Madison, WI), or spectral karyotyping (Cytogenetics & Microscopy Core, NHGRI, NIH), or Illumina Global Screening Array, identity test (Fluidigm SNP Trace Panel, and pluripotency (by FACS and/or immunocytochemistry).

Relevant information on individual iPSC clones will be available in a searchable web portal (https://nimhnetprd.nimh.nih.gov/AMBIGEN/ipscqc) that will go live and accessible once characterization of the first set of ~42 iPSC clones is completed.

Methods used for differentiating iPSCs into NPCs, astrocytes and neurons are described under Supplementary Information.

## Results

### AMBiGen family collection

As of Spring 2022, we recruited and clinically phenotyped 1134 study participants from 407 families in North America and Brazil. Of these, 44% self-identify as Amish and 40% as Mennonite, while the rest represent other or mixed Anabaptist ancestry. Over half of participants have been assigned a Best Estimate diagnosis, yielding the diagnostic breakdown shown in Table 1. Among the US participants, >60% were diagnosed with BPI, schizoaffective bipolar disorder, BPNOS, BPII, MDDR, MDD and SCZ.

**Table 1:** Diagnosis and gender of AMBiGen (US and Brazil) participants.

	US Participants			Brazilian Participants		
Diagnosis	Total	Male	Female	Total	Male	Female
BP I	276	156	120	10	6	4
BP II	81	35	46	8	3	5
Schizoaffective Bipolar Depressed	6	4	2	0	0	0
Schizoaffective Bipolar Manic	24	12	12	0	0	0
Bipolar, Not Otherwise Specified	14	6	8	11	5	6
Major Depressive Disorder, Recurrent (MDDR)	110	37	73	22	6	16
MDD, Single Episode	62	23	39	13	5	8
Schizophrenia	14	8	6	1	1	0
Other Diagnosis	70	32	38	13	3	10
Social Phobia^a^	1	1	0	10	3	7
No Axis I Diagnosis	286	137	149	102	43	59
TOTAL	944	451	493	190	75	115

^a^Listed only when none of the other diagnoses shown are present.

**Fig. 1:**
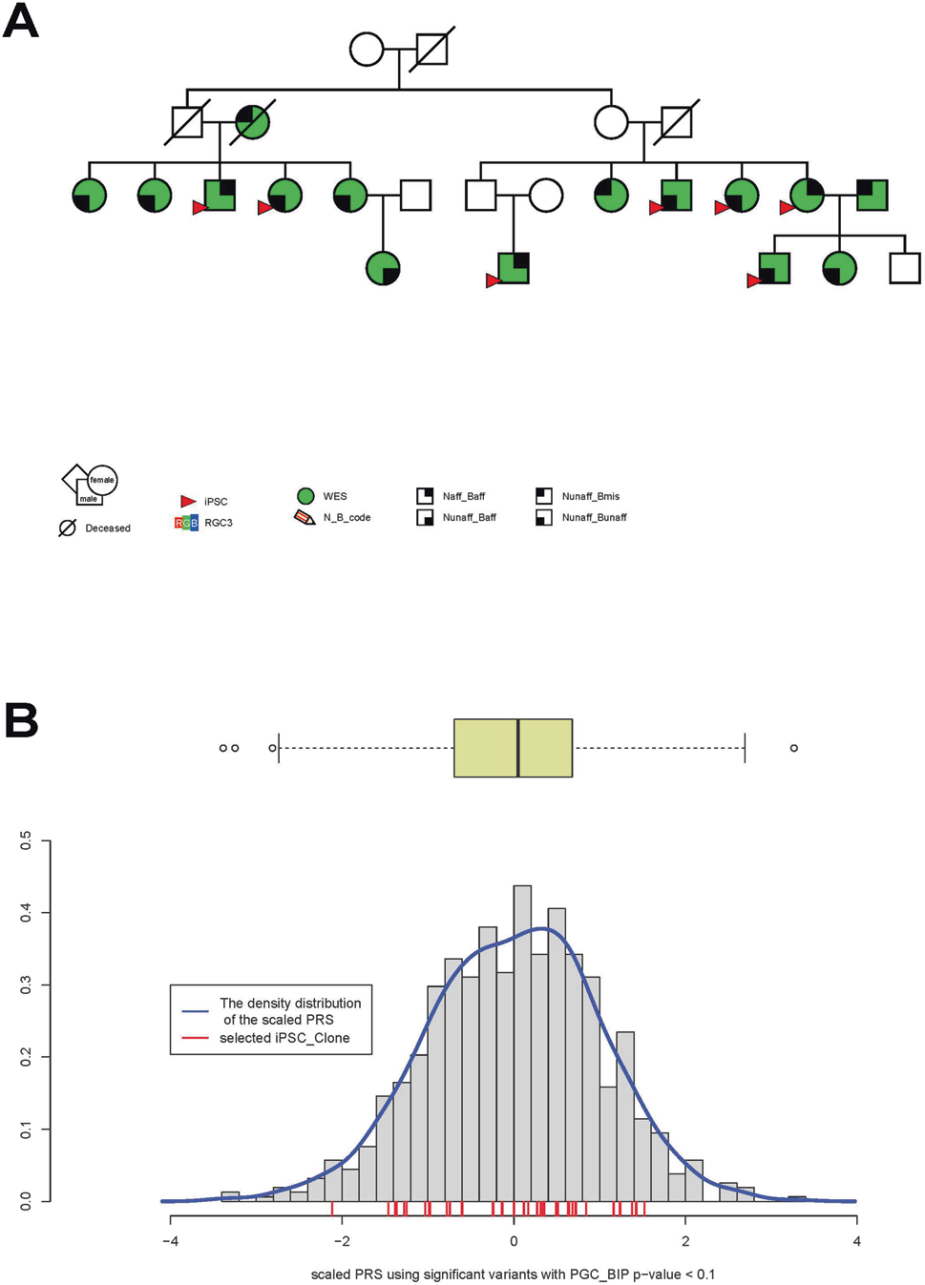
A family branch of the multigenerational Amish pedigree and polygenic risk score (PRS) of study participants. **A** A family branch of the multigenerational Amish pedigree. Green squares and circles indicate participants whose gDNA has been exome sequenced and red triangles indicate those family members for which iPSCs have been generated. Narrow affection status includes BPI and schizoaffective bipolar disorder, and broad affection status includes BPI, BP-II with single/recurrent depression, schizoaffective manic/bipolar/depressed, SCZ, BP-NOS and MDD recurrent. Naff narrow affected, Baff broad affected, Nunaff Narrow unaffected, Bunaff broad unaffected, Bmiss broad missing, WES whole exome sequenced. **B** PRS of study participants. The red bars indicate PRS values for iPSC donors. The box plot depicts the PRS quartiles, and circles outside the box plot represent outliers.

Figure 1A shows a family branch, drawn using Cranefoot 3.2.3 [65], that includes ascertained members of the multigenerational Amish pedigree. As indicated, exome sequencing has been done on 15 members whose affection status has been determined. iPSC clones have been derived from seven family members. The large extended pedigree as shown in the AGDB database [40] is presented in Supplementary Figure 1.

### Enriched rare variants in genes located within BD and SCZ GWAS regions

Whole exome sequencing of gDNA from the initial sample of 324 Amish and Mennonite study participants revealed 7,790 SNVs with MAF ≤ 0.01 shared by at least two individuals. Probable risk genes are those located within 10 kb upstream or downstream of a genome-wide significant GWAS locus for BD [1, 2], SCZ [4, 5], and those ranked in the top 10 by SCHEMA [19] (Table 2, Supplementary Table 1). Within 89 genes, exome sequencing uncovered 112 rare nonsynonymous and protein-disrupting variants (Table 2, Supplementary Table 1).

**Table 2:** Enriched rare variants in AMBiGen genetic isolate.

Cytogen location	Position (hg38)	SNP	Ref/alt allele	Gene^a^	TWAS/SMR; TWAS ATLAS	CADD PHRED	Minor Allele Frequency			Enrichment	
							AMBiGen adjusted for relatedness	AVS	gnomAD non-Finnish European	AVS	gnomAD
2p16.1	58229827	rs143819820	C/G	*FANCL*	Yes	22.6	3.80E-03	4.48E-03	7.93E-04	0.85	4.79
2q11.2	96816839	rs940116018	C/G	*CNNM3*	Yes	22.6	2.30E-03	4.27E-04	–	5.39	–
2q11.2	96839596	rs774867231	G/GGATGCCGCGCT	*ANKRD23*^*b*^	Yes	–	4.20E-03	1.46E-02	2.84E-05	0.29	147.89
2q33.1	197460047	rs375172756	A/G	*COQ10B*	Yes	27.0	3.80E-03	4.05E-03	–	0.94	–
3p22.2	36832166	rs200052241	T/G	*TRANK1*	Yes	21.7	2.90E-03	–	2.83E-04	–	10.24
3p21.1	52440068	rs140387468	G/A	*SEMA3G*	Yes	23.8	4.00E-03	3.78E-03	1.74E-04	1.06	23.01
3p21.1	52506179	rs983375651	G/C	*STAB1*	Yes	24.7	3.20E-03	2.03E-02	–	0.16	–
3p21.1	52711866	rs147140852	DEL/T	*NEK4*^*b*^	Yes	-	2.90E-03	4.32E-03	5.40E-05	0.67	53.75
6p22.2	26240735	–	T/C	*HIST1H4F (H4C6)*^*c*^	–	18.2	2.90E-03	4.83E-03	–	0.60	–
8p21.1	27516387	–	C/G	*EPHX2*	–	24.6	2.90E-03	–	–	–	–
8p11.23	38418290	rs200776757	C/A	*FGFR1*	Yes	23.5	2.70E-03	–	8.21E-04	–	3.29
8q24.3	143985946	–	G/A	*PARP10*	–	22.8	2.90E-03	5.38E-04	–	5.39	–
10q25.1	110112892	rs139343347	C/T	*ADD3*	Yes	23.0	2.70E-03	6.84E-03	1.15E-04	0.39	23.58
11q13.1	65618843	rs893253614	C/T	*PCNX3*	Yes	22.7	4.20E-03	1.80E-02	–	0.23	–
11q13.1	66043272	rs774640662	G/T	*GAL3ST3*	Yes	23.1	3.80E-03	3.12E-03	9.09E-06	1.22	418.09
11q24.3	130903357	rs1043508580	G/C	*SNX19*	Yes	27.8	5.10E-03	4.32E-03	8.81E-06	1.18	579.15
12q24.3	57098559	rs200507162	G/A	*STAT6*	Yes	21.7	2.90E-03	1.70E-03	8.80E-06	1.71	329.62
12q24.31	122982523	rs771187096	C/G	*ARL6IP4*	Yes	24.7	3.80E-03	3.83E-03	9.00E-06	0.99	422.46
12q24.31	123176727	rs777110388	C/T	*MPHOSPH9*	Yes	23.3	2.90E-03	5.38E-04	–	5.39	–
15q15.2	42729236	rs779110523	C/T	*CDAN1*	Yes	23.1	3.10E-03	–	2.65E-05	–	116.89
15q25.3	84798646	rs987445362	A/G	*ZNF592*	Yes	23.8	4.20E-03	4.64E-03	–	0.91	–
15q25.3	84840736	rs150985765	C/T	*ALPK3*	Yes	25.8	2.90E-03	4.86E-03	1.59E-04	0.60	18.28
15q26.1	90881727	rs35641241	G/A	*FURIN*	Yes	24.0	2.70E-03	–	1.17E-03	–	2.32
15q26.1	90887034	rs372642630	G/A	*FES*	Yes	31.0	2.10E-03	–	7.05E-05	–	29.77
16p11.2	30092741	rs200869087	T/G	*YPEL3*	Yes	22.9	2.70E-03	2.45E-03	1.50E-04	1.10	17.99
16q21	58587853	rs142749838	G/A	*CNOT1*	Yes	22.8	4.90E-03	1.14E-02	4.93E-04	0.43	9.94
16q22.1	67675340	rs146594195	G/A	*GFOD2*	Yes	24.2	3.70E-03	2.21E-02	1.41E-04	0.17	26.19
16q22.1	67968618	rs563719406	C/T	*SLC12A4*	Yes	20.7	2.90E-03	5.12E-03	6.97E-04	0.57	4.16
16q22.1	68078793	rs148640115	G/A	*DUS2*	Yes	27.4	2.10E-03	–	9.07E-04	–	2.32
17q21.1	40033408	rs374332490	A/G	*MED24*	Yes	28.0	3.20E-03	1.62E-03	1.82E-05	1.98	176.21
19p13.11	19492319	rs200305535	G/A	*GATAD2A*	Yes	29.3	2.10E-03	7.03E-03	7.55E-04	0.30	2.78
20q13.12	38771664	rs1306410634	G/A	*ACTR5*	Yes	28.6	3.20E-03	–	8.79E-06	–	364.01

^a^Mutation is nonsynonymous unless otherwise indicated.

^b^Frameshift insertion.

^c^Stoploss.

The shorter list of variants in Table 2 includes 32 of the 78 variants that were enriched >2-fold, after adjusting for relatedness, when compared to gnomAD reference sample of unrelated non-Finnish Europeans (Table 2, Supplementary Table 1). High levels of enrichment over gnomAD MAF were detected: >500-fold enrichment in *SAPCD1* and *SNX19*, one allele of *SYNE1* and variants in *GAL3ST3* and *ARL6IP4* were enriched >400 fold, and alleles enriched >300 fold were carried by *ALAS1*, *ITIH1*, *DOPEY1*, *SPTBN2*, *STAT6*, *ACTR5* and *ADRM1*. Further studies are needed to clarify the role of variant enrichment in disease risk in this sample.

Three new ultrarare nonsynonymous variants that have not been assigned yet to known SNPs were revealed in the AMBiGen sample. Since the rare variant in *EPHX2* (8p21.1) was not detected in either AVS or gnomAD, we designated it as a private variant (Table 2). A novel rare variant in *PARP10* (8q24.3), was absent in gnomAD but was >5-fold enriched in our sample compared to AVS. A third ultrarare new allele creates a stop-loss mutation in *HIST1H4F* (*H4C6*) (6p22.2), was absent in gnomAD and displayed a lower frequency in AVS than in our AMBiGen sample (Table 2).

In contrast to the ultrarare variants within the genes that had no associated SNP, the ultrarare variant in *DXO* (6p21.33), which was absent in both AVS and gnomAD, is an allele of a known SNP, rs371065709 (Supplementary Table 1). Thus, this variant may qualify also as a private variant in AMBiGen (Table 2). Some rare nonsynonymous variants are represented at lower frequencies in AMBiGen than in AVS. This is expected given the differences in representation of various Anabaptist demes across the AVS samples (Table 2, Supplementary Table 1).

The difference in genetic background between AVS and gnomAD is unmasked further by the fact that the 16 rare variants in this AMBiGen sample that were absent in gnomAD did not match the 20 rare alleles that were missing in AVS (Table 2, Supplementary Table 1).

### Potential associated function of rare variant carrying genes and variant deleteriousness

To determine whether any of the genes that carry rare variants might be functionally relevant to neuropsychiatric phenotypes, we searched published TWAS/SMR studies. In TWAS/SMR published reports [2, 26, 29, 58], 28 of the 89 rare variant carrying genes have been shown to undergo dysregulation (Table 2, Supplementary Table 1). We also searched the TWAS Atlas [59], which revealed an additional seven associated genes (Table 2, Supplementary Table 1). Gene-trait association was mostly seen with SCZ, additionally, *TRANK1*, *ADD3* and *CDAN1* were TWAS positive for BD, *OSBPL3* for depressive disorder and *CACNA1G* for ADHD.

Variant deleteriousness reflected in the CADD_PHRED scores [60, 61] revealed that of the 112 rare nonsynonymous variants, four had a CADD score of >30 while 61 other variants had a score of >20 (Table 2, Supplementary Table 1). Variants for *ITIH1* and *RPJL* showed the highest CADD-PHRED score of 40, and *DPP3* and *FES*, each have a score of 31, suggesting a high level of deleteriousness.

### Rare CNVs in the AMBiGen sample

**Fig. 2:**
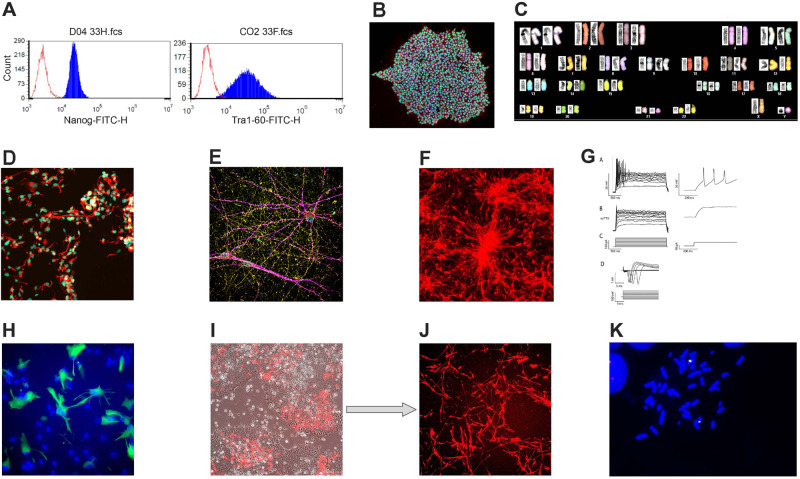
Analysis of AMBiGen-derived iPSCs and iPSC neural derivatives. **A** FACS analysis indicating pluripotency of iPSC clone using Nanog and Tra1-60 markers. **B** Immunocytochemistry with Oct4 (green) and SSEA4 (red) pluripotency markers and DAPI. **C** Spectral karyotyping of iPSC clone indicating normal 46,XY karyotype. **D** NPCs bound to nestin (red) and PAX6 (green) markers and DAPI. **E** Neurons, 8 weeks post differentiation showing binding to synaptophysin (red), MAP2 (purple) and PSD95 (green) markers. **F** Neurons differentiated from NPCs labeled with tdTomato. **G** Spontaneous neuron action potential. **H** iPSC-derived astrocytes binding to GFAP marker. **I** Direct differentiation: iPSC transfected with hNGN2 plasmid, with mCherry. **J** hNGN2 transfected iPSC differentiated to neurons upon doxycycline treatment. **k** FISH showing heterozygous duplication on 16p11.2.

Whole genome SNP array detected recurrent rare CNVs on 1p36.33, 15q13.3, 16p11.2, 16p12.2, and 22q11.2, all overlapping with those shown to be pathogenic in neuropsychiatric disorders [10–15] (Table 3). This list includes samples from unrelated families with reciprocal duplication and deletion CNVs on 16p11.2. Duplication on 16p11.2 was further shown through fluorescence in situ hybridization using a BAC probe (Fig. 2k). *YPEL3*, the only gene within the 16p11.2 CNV that was found to carry a rare nonsynonymous allele, was enriched ~18-fold in AMBiGen compared to gnomAD (Table 2).

**Table 3:** Amish and Mennonite iPSCs.

Rutger’s ID	iPSC Clone ID	Sex	Age	Diagnosis	Exome Sequence^b^	Neuropsychiatric CNV	CNV State	Rutger’s ID	iPSC Clone ID	Sex	Age	Diagnosis	Exome sequence	Neuropsychiatric CNV	CNV State
10C117904	HT416	F	52	Schizoaffective Manic	Yes	16p11.2	Duplication	MH0263946	HT881^a^	M	71	BP-II w. MDD, single episode	No		
MH0255672	HT839^a^	F	18	Schizophrenia	Yes	22q11.2	Deletion	NA	HT281	F	28	Adjustment Disorder	No		
MH0212842	HT440	M	35	MDD, Single episode	Yes			MH0197466	HT477	M	58	BP NOS	No		
MH0138647	BP006	M	24	BP-I	Yes			MH0263940	HT879^a^	M	33	Hypomania	No		
09C96459	BP005	M	23	BP-I	Yes			MH0231416	HT908^a^	F	20	MDD, Single episode	Yes	16p11.2	Deletion
09C96359	HT33	M	21	BP-I	Yes			NA	HT444	F	92	No Best Estimate	Yes		
MH0137345	HT35	F	42	BP-I	Yes			MH0183286	HT473	F	65	No Best Estimate	No		
MH0130482	HT278	M	68	BP-I	Yes			MH0217921	HT34	M	30	No Axis I Diagnosis	Yes		
MH0148732	HT280	M	22	BP-I	Yes	16p12.2	Duplication	MH0151313	HT36	F	46	No Axis I Diagnosis	Yes	1p36.3	Duplication
MH0202383	HT439	M	26	BP-I	Yes			NA	HT279	M	71	No Axis I Diagnosis	No		
MH0132281	HT441	M	35	BP-I	Yes			MH0180067	HT417	F	59	No Axis I Diagnosis	No		
MH0146073	HT443	F	86	BP-I	Yes			NA	HT442	M	35	No Axis I Diagnosis	No		
MH0156979	HT445	M	36	BP-I	Yes			MH0168485	HT446	M	30	No Axis I Diagnosis	No		
MH0171056	HT448	M	21	BP-I	Yes			MH0198838	HT447	M	19	No Axis I Diagnosis	No		
MH0196692	HT450	M	40	BP-I	Yes			MH0196697	HT449	F	48	No Axis I Diagnosis	No		
10C114252	HT466	M	37	BP-I	Yes			MH0218686	HT467	M	40	No Axis I Diagnosis	Yes		
MH0151314	HT470	M	61	BP-I	Yes			MH0197448	HT468	M	35	No Axis I Diagnosis	Yes		
MH0159996	HT471	F	56	BP-I	Yes			MH0177505	HT475	M	74	No Axis I Diagnosis	No		
MH0152565	HT472	F	36	BP-I	Yes			MH0174506	HT613	M	60	No Axis I Diagnosis	Yes	16p11.2	Duplication
MH0177506	HT474	M	72	BP-I	Yes			MH0235414	HT615	F	60	No Axis I Diagnosis	Yes		
MH0185455	HT476	M	18	BP-I	No			MH0141461	HT616	F	80	No Axis I Diagnosis	Yes		
MH0202331	HT614	M	62	BP-I	Yes			MH0224715	HT620	F	69	No Axis I Diagnosis	No		
MH0162693	HT619	F	61	BP-I	Yes			MH0138651	HT622	F	63	No Axis I Diagnosis	Yes		
MH0141646	HT623	F	19	BP-I	Yes			MH0223905	HT624	M	26	No Axis I Diagnosis	Yes		
MH0168295	HT658	F	33	BP-I	Yes			MH0218170	HT659	F	32	No Axis I Diagnosis	Yes		
MH0263617	HT878^a^	M	48	BP-I	No			MH0255662	HT838^a^	F	20	No Axis I Diagnosis	No	15q13.3	Duplication
MH0263948	HT882^a^	M	62	BP-I	No			MH0263942	HT880^a^	M	67	No Axis I Diagnosis	No		
MH0151307	HT469	M	21	BP-II w. MDDR	Yes			MH0263608	HT883^a^	F	38	No Axis I Diagnosis	No		
MH0139613	HT621	F	38	BP-II w. MDDR	Yes			MH0269303	HT909^a^	M	20	No Axis I Diagnosis	No	16p11.2	Deletion
MH0197488	HT657	F	40	BP-II w. MDDR	No			MH0269317	HT910^a^	M	25	No Axis I Diagnosis	No		
MH0231414	HT907^a^	M	48	BP-II w. MDDR	Yes	16p11.2	Deletion								

^a^PBMC as somatic cell source, all others were derived from fibroblast lines.

^b^No: in the process of being sequenced.

### Polygenic risk scores

To evaluate the cumulative risk for BD caused by common variants in the genome, we calculated PRS in the AMBiGen sample, including all iPSC donors. The PRS values based on SNPs with PGC BIP p-value threshold < 0.1 were scaled and plotted by density (Fig. 1B). PRS for most of the study participants are indicated by the red bars on the X-axis (Fig. 1B). iPSC clones are available within all four quartiles of the PRS distribution and extreme outliers were not detected, most likely due to sample relatedness.

### Creation and characterization of iPSC resource

To date, our growing iPSC collection includes clones from 61 genetic isolate donors, approximately half are diagnosed with a major mood disorder, of which 24 have BP-I diagnosis (Table 1, Table 3). SNP array analysis on iPSC clones revealed five neuropsychiatric CNVs (1p36.33 dup, 15q13.3 dup, 16p11.2 del, 16p11.2 dup, and 22q11.2 del) across 10 distinct donors (Table 3).

iPSCs that are selected and banked for downstream experiments show the following features: (a) genotype corresponds to that of the cell-of-origin (Supplementary Table 2), (b) pluripotent (Fig. 2A, B), (c) normal karyotype (Fig. 2C), (d) can be differentiated into cell type of interest; e.g., neural derivatives (Fig. 2D–J), (e) reasonable growth rates (doubling time ~24 hours), (f) no visible evidence of substantial spontaneous differentiation, and (g) no evidence of mycoplasma and other culture contamination (Supplementary Table 2).

We continue our on-going effort to generate iPSC clones from our expanding sample collection and we are developing a searchable web portal (https://nimhnetprd.nimh.nih.gov/AMBIGEN/ipscqc) that links relevant data for individual iPSC clones, e.g., somatic cell source, characterization, and quality control. An example of the entries is presented in Supplementary Table 2. Investigators can search for iPSC clones of interest. Clones will be banked at Rutgers Cell & DNA Repository (Infinite Biologics), which will distribute iPSC clones to qualified investigators. Individual-level phenotype and genotype data is available via dbGAP (phs000899).

## Discussion

This research project has the overarching goal of contributing to the understanding of the genetic etiology and underlying biology of BD and related neuropsychiatric disorders. We have ascertained, clinically phenotyped, and genomically characterized participants drawn from genetically isolated Anabaptist populations. As part of this work, we have generated and characterized a unique resource of human iPSC lines from affected participants and their relatives. This iPSC resource that will be made available to the research community provides a sustainable repository of human-derived stem cells for studies that aim to model BD in vitro. We hope these studies demonstrate how specific genetic variants alter neurobiological mechanisms that lead to disease. Such studies may also uncover molecular targets for therapeutic interventions.

To determine the genomic architecture of the sample collection, some of which were selected for the iPSC resource, we performed whole exome sequencing and whole genome SNP array genotyping. Analysis of these data yielded diverse rare nonsynonymous, and protein-disrupting alleles in genes within GWAS loci whose allele frequencies are enriched in this sample when compared to general (gnomAD) and Anabaptist-based reference samples. The role of these enriched rare, potentially functional alleles in neuropsychiatric risk is not yet clear and awaits further investigation.

We highlight three novel ultrarare variants, absent in dbSNP, that were identified in the genetic isolates and need to be validated. A private variant detected in *EPHX2* at chr8:2751687 (Table 2), creates an amino acid substitution, Ser300Cys (NM_001979) (UCSC Genome Browser, GENCODE V41). Prior reports have shown that expression of the epoxide hydroxylase 2 (EH) protein was significantly higher in MDD, BD and SCZ parietal cortex and liver than in controls [66, 67]. A small study has reported that lipid metabolism mediated through soluble EH activity was associated with winter depression in patients with seasonal affective disorder [68].

Another ultrarare nonsynonymous allele is displayed by *HIST1H4F* (*H4C6*) which encodes histone 4, one of four histone components of nucleosomes. The variant causes a loss of the stop codon, TGA, which is replaced by CGA that codes for arginine, giving rise to Ter104Arg (NM_003540) (UCSC Genome Browser, GENCODE V41). The mutation might result in an abnormal elongation of the polypeptide chain leading to a possible disruption of nucleosome structure and function.

*PARP10* displayed an ultrarare new variant, a 3’G > A5’ (5’C > T3’) change creating the missense mutation, H71M (NM_032789) (UCSC Genome Browser, GENCODE V41). The variant is in exon 3, which encodes the RNA recognition motifs 1 & 2 (UCSC Genome Browser, GENCODE V41).

We also show in this study several loci in GWAS regions that are TWAS positive and thus might be considered when prioritizing genes that may be causal of neuropsychiatric phenotypes. In a recent study of a population cohort of >90,000 that included adult patients with ASD, BD and SCZ, >90 genes were shown to carry rare, loss-of-function, pathogenic variants [69]. Included in this group were *SCN2A*, *TCF20*, and *PRR12*, genes that showed enriched rare mutations in our sample (Supplementary Table 1). However, mutations identified in our study, *SCN2A* (p.E318K) and *TCF20* (p.S1803A) (UCSC Genome Browser) did not overlap with those reported by Shimelis et al. [69], i.e., *SCN2A* (p.Arg1626Ter) and *TCF20* (p.Ser513CysfsTer8). Whether any of these mutations contribute to psychiatric phenotypes in AMBiGen remains to be investigated.

Several study participants carried rare CNVs that overlapped with known pathogenic neuropsychiatric CNVs. An apparently de novo 22q11.2 deletion was detected in a proband with schizophrenia, short stature, and intellectual disability. All three carriers of the 16p11.2 duplication were found within the same nuclear family. The proband has schizoaffective bipolar disorder, her carrier son has MDD and mild intellectual disability, and her carrier brother was psychiatrically and cognitively healthy when evaluated at age 60. All three carriers of the 16p11.2 deletion also belong to the same nuclear family. The carrier father has BPI and mild intellectual disability, while his carrier daughter has MDD with normal cognition. A carrier brother has declined psychiatric assessment. The 1p36.3 duplication is seen in a psychiatrically unaffected woman who married into a large pedigree with several cases of BD but no known pathogenic CNV. The 15q13.3 duplication was found in the unaffected grandmother of a proband with schizophrenia who has not yet undergone CNV screening.

There are several important limitations in this study. The sample size remains underpowered to detect association with any but the most penetrant rare alleles, although power is increased when otherwise rare alleles are enriched through genetic drift. In addition, AMBiGen derives from multiple founder populations with many distinctive variant enrichments not perfectly represented in AVS. Many such variants are rare in the broader population, but many population-enriched alleles have not been shown to be associated with disease. It is plausible that in more recently isolated populations some enriched, rare variants, could exert large effects on risk for BD. Although we have presented findings of enriched rare, potentially deleterious variants, we hasten to add that at this stage of our study, given the current sample size, there is no evidence that such variants confer a role in susceptibility to BD in AMBiGen, therefore functional validation is premature. It is important to emphasize that adequately powered association of any variant will require recruitment of additional carriers. We are currently seeking to extend pedigrees in which probands carry otherwise rare, loss of function variants. This is much more labor-intensive than genotype-first call back studies.

To establish cause-and-effect in disease, the biological mechanisms perturbed by underlying genetic variations need to be established. Toward this goal, we are pursuing iPSC-based in vitro modeling studies enabled through this iPSC resource. So far, iPSC lines have been generated from 61 donors, many of whom are diagnosed with BD and related neuropsychiatric illnesses. To expand the resource, we are continuing to reprogram additional donor somatic cells and characterizing resultant iPSC clones.

Pending expansion of this iPSC resource, currently we are not pursuing studies that aim to contrast samples with very high versus very low burdens of risk since, so far, the range of PRS observed among related individuals in AMBiGen is relatively narrow. On the other hand, each iPSC line from an affected participant is well matched genetically by one or more unaffected relatives, which should facilitate studies of highly penetrant alleles and CNVs.

iPSC-based studies involve time-consuming, multi-step processes, that demand care starting from sample collection, somatic cell isolation, reprogramming, subsequent steps that include cell culture, clone characterization, differentiation into disease-relevant derivatives and functional genomic assays. Future approaches in multiplexing and development of standardized, high-throughput, efficient and automated techniques would be beneficial. Complementing monolayer with a 3D brain organoid platform [70] could help model temporal and spatial aspects of neural development, maturation and role in disease of various brain anatomical structures and cell types, although circuitry and vascularization remain to be incorporated adequately in the structural network.

iPSC-based models cannot fully recapitulate the hallmarks of neuropsychiatric diseases; however, they provide a renewable cellular reagent to examine disease-associated alterations in genomic, cellular, epigenetic, and molecular landscape of diverse neural cell types at various temporal stages. In addition, iPSC-based models permit a systematic interrogation of the dynamic effects of medications, biologic insults, and environmental stressors (Supplementary Fig 2).

This collection of iPSC lines from clinically and genomically well-characterized participants drawn from genetically isolated populations will provide a unique resource for future studies. To promote accessibility to the research community, we have developed a searchable web portal (https://nimhnetprd.nimh.nih.gov/AMBIGEN/ipscqc) that contains relevant information for each iPSC clone and its corresponding donor. Clinical and genetic data for each of the donors are deposited in dbGaP. These on-line databases will help interested investigators select iPSC clones that would be useful for studies that may help reveal causal genes and signature pathways for BD and related neuropsychiatric disorders.

### Supplementary information

Supplementary Information

Table S1

Table S2

Figure S1

Figure S2
