# Supplementary material for: A resource of induced pluripotent stem cell (iPSC) lines including clinical, genomic, and cellular data from genetically isolated families with mood and psychotic disorders
Source: Transl Psychiatry. 2023 Dec 16;13:397. doi: 10.1038/s41398-023-02641-w (PMC10725500; doi:10.1038/s41398-023-02641-w)
Supplement: Supplementary file 1 — Supplementary Information [file 41398_2023_2641_MOESM1_ESM.docx]

**SUPPLEMENTARY INFORMATION**

**Details of ascertainment, clinical assessment, and phenotyping**

Most of the patient recruitment came from various treatment facilities. Plain communities receive psychiatric care in a variety of settings, but many prefer to use providers that accept uninsured patients and settings deemed culturally appropriate for Anabaptists. Since 2009, we have developed cooperative relationships with several such providers who advertise the AMBiGen study to patients and help those interested make initial contact with a study representative. Cooperating providers include inpatient facilities such as Green Pasture and Sunrise Meadows (PA), and Horizons of Hope, Pleasant Haven, and Rest Haven (IN) that provide supervised living for extended stays or a transition from hospital to home.

We also recruit directly from the communities by using advertisements in publications aimed at Anabaptist readers (e.g., Die Botschaft and The Mennonite). In addition, we seek to engage communities through educational presentations at churches, meeting halls, information booths at auctions and other public events, visits to elders and other community leaders, in-service presentations for providers at treatment facilities, and contact with mental health support groups. Developing a strong relationship with this population and building trust requires considerable traveling and in-person communication.

**Prescreening and enrollment**

***Eligibility Criteria and Prescreening***

One of the major goals of this project is to collect a large sample of affected sibling pairs, cases, and extended families with familial bipolar and related disorders. To be eligible for this study an individual needs to be diagnosed with BD or related disorders. Nuclear family and extended relatives of the affected proband are also eligible whether they are unaffected or affected with a disorder. The study uses DSM-IV and 5 criteria for the definition of ‘affected.’ All participants must be over the age of 18 and be able to provide informed consent. In addition, they must be willing to give a biological sample and participate in an interview. Any individual that engaged in active alcohol or substance use is excluded. All participants go through a prescreening process to ensure they meet the eligibility criteria either in-person or over the phone. If a participant is deemed to be ineligible, all recorded information is discarded. Once an individual is determined to be eligible, a clinician will work with the participant to go over the consent form to ensure they understand all aspects of the study.

***Informed consent***

To participate in the study, every volunteer must complete an IRB-approved written consent that covers the study background, objectives, procedures, expectations, confidentiality, reimbursement, and opt out options. Volunteers have the option to consent to certain study procedures, which include giving permission to contact relatives, agreeing to have their anonymized data shared with other researchers, and providing the release of medical records. Volunteers who agree to have their DNA sequenced are also asked whether they would like to be informed of any actionable incidental findings that may arise.

***iPSC Quality Control (QC)***

For every reprogrammed somatic cell, the iPSC Core returns up to four iPSC clones, each with three replicates. To check for pluripotency, the Core performs fluorescence-activated-cell-sorting (FACS) on two lines with Nanog and Tra-1-60 markers (Fig. 2A). We conduct immunocytochemistry (ICC) using the same or other (e.g., SSEA4 and Oct4) pluripotency markers (Fig. 2B). iPSCs are processed as described previously [1] using either E8 (ThermoFisher Scientific, MA) or mTeSR1 (STEMCELL Technologies, Canada) as growth medium.

Pluripotent iPSCs are selected for further processing based on criteria the following criteria: no more than minor levels of spontaneous differentiation, absence of contamination and reasonable growth rate with a doubling time of approximately 24 hours. Clones that meet these criteria are sent for karyotyping, either to the Cytogenetics and Microscopy Core, National Human Genome Research Institute, NIH, or to WiCell (Madison, WI). Two or more clones of each iPSC line that display a normal karyotype are picked for further studies (Fig. 2C). Clones that pass QC are expanded, passaged, resuspended in Cryostor CS10 freezing medium, transferred to cryovials that are then barcoded, and stored in liquid nitrogen (vapor phase).

To recover frozen clones, iPSCs are thawed and grown overnight in iPSC medium with a supplement of either 10 µM Rock Inhibitor (RI) or 1x RevitaCell (RC) (Thermo Fisher Scientific, MA). The following day medium exchange with fresh medium without either RI or RC is done; henceforth, daily exchange with fresh medium is performed.

De-identified clinical data, DNA samples, and iPSC clones are freely available to researchers through public repositories (dbGAP phs000899.v1.p1 NIMH Bipolar Amish).

***Differentiation into neural cells***

***iPSCs into NPCs***

iPSCs are sub-cultured up to ≥15 passages to deplete Sendai virus. Differentiation into NPCs is initiated following the neural rosette method (STEMCELL Technologies, Canada). NPCs are grown to confluency, passaged at high density, expanded, resuspended in NPC freezing medium (STEMCELL Technologies, Canada), cryovials barcoded and banked in liquid nitrogen. Typically, positive binding of neural stem cell markers such as nestin and PAX6 in ICC indicates production of NPCs (Fig. 2D).

***NPCs into neurons***

NPCs are differentiated into neurons in a cocktail consisting of a basal medium, BrainPhys (STEMCELL Technologies) or Neurobasal (Thermo Fisher Scientific, MA) supplemented with BDNF, GDNF, B-27-without vitamin A, N2, dbcAMP, ascorbic acid and laminin [2]. Neuron differentiation is maintained up to ≥ four weeks with medium changes once every few days (Fig. 2E). A mixed population of forebrain-type neurons is generated and antibodies to MAP2, synaptophysin, and PSD95 are used for ICC.

To permit live-cell fluorescence imaging, NPCs may be labeled with tdTomato [3], before differentiation into neurons. Fluorescently labeled NPCs are expanded and banked to provide a sustainable supply for downstream studies. During differentiation, the neuron cell body and neurites, as well as astrocytes, retain the red fluorescence of labeled NPCs, enabling clear visualization of live cellular processes in real-time (Fig.2F). We also have performed electrophysiology by patch clamp on selected lines to test for neuronal action potentials (Fig. 2G).

***NPCs into astrocytes***

NPCs are differentiated into astrocytes following the protocol described previously [4] and passaged until cells show positive binding to S100β (calcium-binding beta protein, primarily expressed in astrocytes) and GFAP (glial-fibrillary acidic intermediate filament protein) antibodies. Barcoded stocks of GFAP-positive astrocytes are banked until further use (Fig. 2H). qRT-PCR for astrocyte-specific markers: *EAAT1*, *ALDOC*, *CX43*, *SOX9* and *NFIA* would further indicate successful astrocyte differentiation [5]. Spontaneous transient calcium activity, a distinct property of astrocytes, can also be measured [6, 7].

***Direct differentiation of iPSC into cortical excitatory neurons (iNs)***

Direct differentiation of iPSCs into stable transgenic neuronal lines has been achieved via transfection with a neurogenin 2 (NGN2) construct tagged with mCherry [8]. mCherry-positive colonies that carry a homozygous insertion of hNGN2 are propagated, expanded (Fig. 2I), resuspended in Cryostor CS10 freezing medium and stored in LN2. Doxycycline treatment of cells expressing NGN2 induces differentiation into neurons. Neurites start to emanate from cells within hours of drug treatment (Fig. 2J). Frozen early-stage neurons are barcoded, banked, recovered, and brought to maturity as needed. The NGN2-based method drives and supports differentiation into a homogeneous population of excitatory glutamatergic neurons. Antibody markers for excitatory neurons include vGLUT1 and vGlut2 [8, 9].

**Searchable databases**

Genome data including SNP array genotypes and exome sequences are deposited in dbGAP (https://www.ncbi.nlm.nih.gov/gap).

We have created and maintained the following in-house databases:

1. Searchable web portal: <https://nimhnetprd.nimh.nih.gov/AMBIGEN/ipscqc>
2. Clinical-Family resource database: includes ID, relationships, affection status, sex, age, place of residence
3. iPSC repository database: organized using FreezerPro software that permits entry of iPSC clone IDs, barcodes, dates, passage number for iPSCs, NPCs, astrocytes and neurons, location/position in liquid nitrogen freezer, information on removal of vials.
4. iPSC characterization database: organized using FileMaker software containing the following entries: clone IDs, pluripotency, karyotype, CNV, iPSC ICC, neural subtypes ICCs, assays, other relevant images.

**Supplementary Tables**

**Supplementary Table 1. Rare variants within genes in BD and SCZ GWAS regions in the genetic isolates.** An expanded list of nonsynonymous and protein-disrupting variants in the AMBiGen sample. Shown are levels of enrichment versus either AVS or gnomAD, CADD-PHRED score for each variant, and TWAS data for each gene.

**Supplementary Table 2. Characterization of iPSC clones.** Shown are examples of data that will be made available for each iPSC line. Clone images will also be made available.

**Supplementary Figures**

**Supplementary Fig. 1: Extended multigenerational Amish pedigree from AGDB.** Individual members that have been ascertained are represented with red-filled circles or squares.

**Supplementary Fig. 2: Interrogation of genetics and neurobiology of BD and related neuropsychiatric disorders in the genetic isolates.** Multi-omic and phenotype assays on iPSC-derived neural cells to identify molecular, epigenetic, and neurobiological perturbations in BD and potential therapeutic targets.

**References**

1. Beers J, Gulbranson DR, George N, et al. Passaging and colony expansion of human pluripotent stem cells by enzyme-free dissociation in chemically defined culture conditions. *Nat Protoc*. 2012;7(11):2029-2040.

2. Brennand KJ, Simone A, Jou J, et al. Modelling schizophrenia using human induced pluripotent stem cells. *Nature*. 2011;473(7346):221-225.

3. Cerbini T, Funahashi R, Luo Y, et al. Transcription Activator-Like Effector Nuclease (TALEN)-Mediated CLYBL Targeting Enables Enhanced Transgene Expression and One-Step Generation of Dual Reporter Human Induced Pluripotent Stem Cell (iPSC) and Neural Stem Cell (NSC) Lines. *PLOS ONE*. 2015;10(1):e0116032.

4. Tcw J, Wang M, Pimenova AA, et al. An Efficient Platform for Astrocyte Differentiation from Human Induced Pluripotent Stem Cells. *Stem Cell Rep*. 2017;9(2):600-614.

5. Mizuno GO, Wang Y, Shi G, et al. Aberrant Calcium Signaling in Astrocytes Inhibits Neuronal Excitability in a Human Down Syndrome Stem Cell Model. *Cell Rep*. 2018;24(2):355-365.

6. Li C, Ou R, Chen Y, et al. Mutation analysis of seven SLC family transporters for early-onset Parkinson’s disease in Chinese population. *Neurobiol Aging*. 2021;103:152.e1-152.e6.

7. Inazu M. Functional Expression of Choline Transporters in the Blood-Brain Barrier. *Nutrients*. 2019;11(10):E2265.

8. Fernandopulle MS, Prestil R, Grunseich C, Wang C, Gan L, Ward ME. Transcription Factor–Mediated Differentiation of Human iPSCs into Neurons. *Curr Protoc Cell Biol*. 2018;79(1):e51.

9. Zhang Y, Pak C, Han Y, et al. Rapid single-step induction of functional neurons from human pluripotent stem cells. *Neuron*. 2013;78(5):785-798.
