# Supplementary material for: A resource of induced pluripotent stem cell (iPSC) lines including clinical, genomic, and cellular data from genetically isolated families with mood and psychotic disorders
Source: Transl Psychiatry. 2023 Dec 16;13:397. doi: 10.1038/s41398-023-02641-w (PMC10725500; doi:10.1038/s41398-023-02641-w)

**Table S2.  Characterization of iPSC clones.** Shown are examples of data that will be made available for each iPSC line.


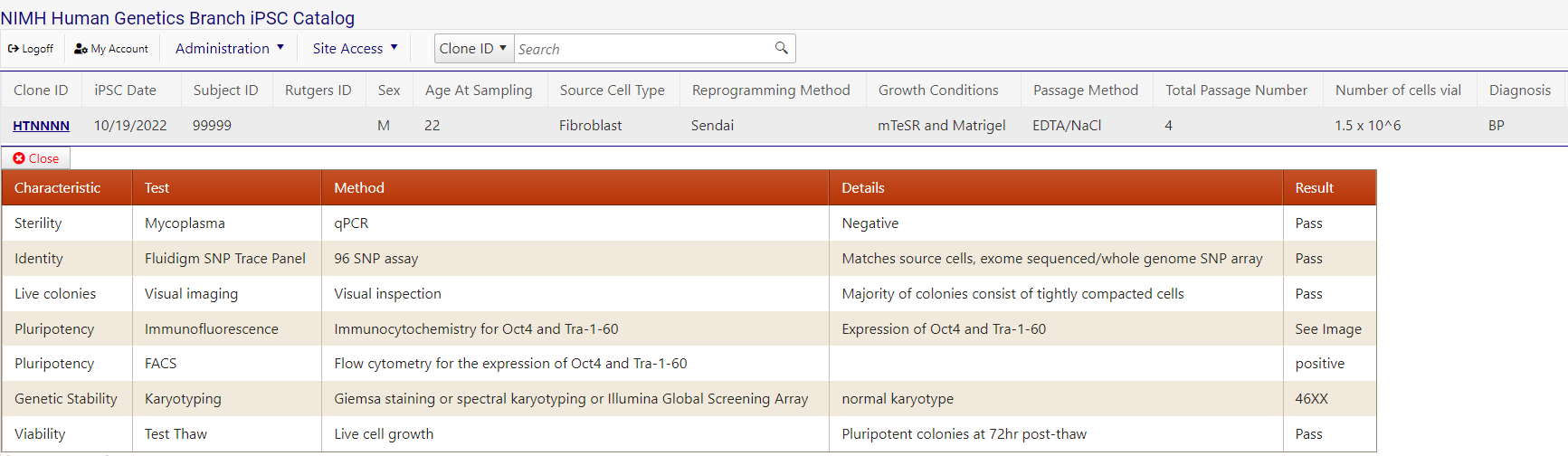

Supplement: Supplementary file 3 — Table S2 [file 41398_2023_2641_MOESM3_ESM.docx]
