# Supplementary figures and images for: A resource of induced pluripotent stem cell (iPSC) lines including clinical, genomic, and cellular data from genetically isolated families with mood and psychotic disorders
Source: Transl Psychiatry. 2023 Dec 16;13:397. doi: 10.1038/s41398-023-02641-w (PMC10725500; doi:10.1038/s41398-023-02641-w)

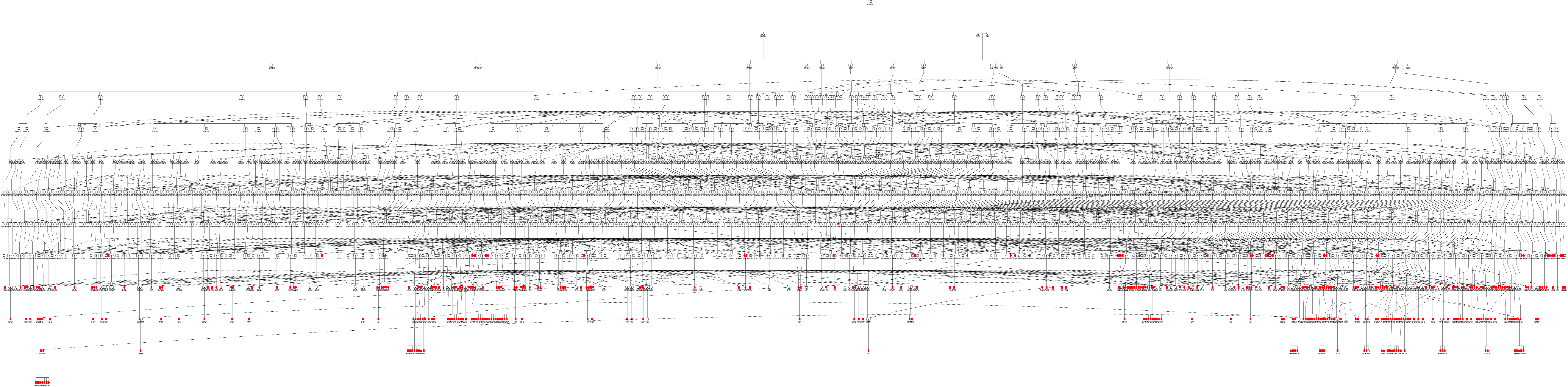

Supplement: Supplementary file 4 — Figure S1 [file 41398_2023_2641_MOESM4_ESM.png]

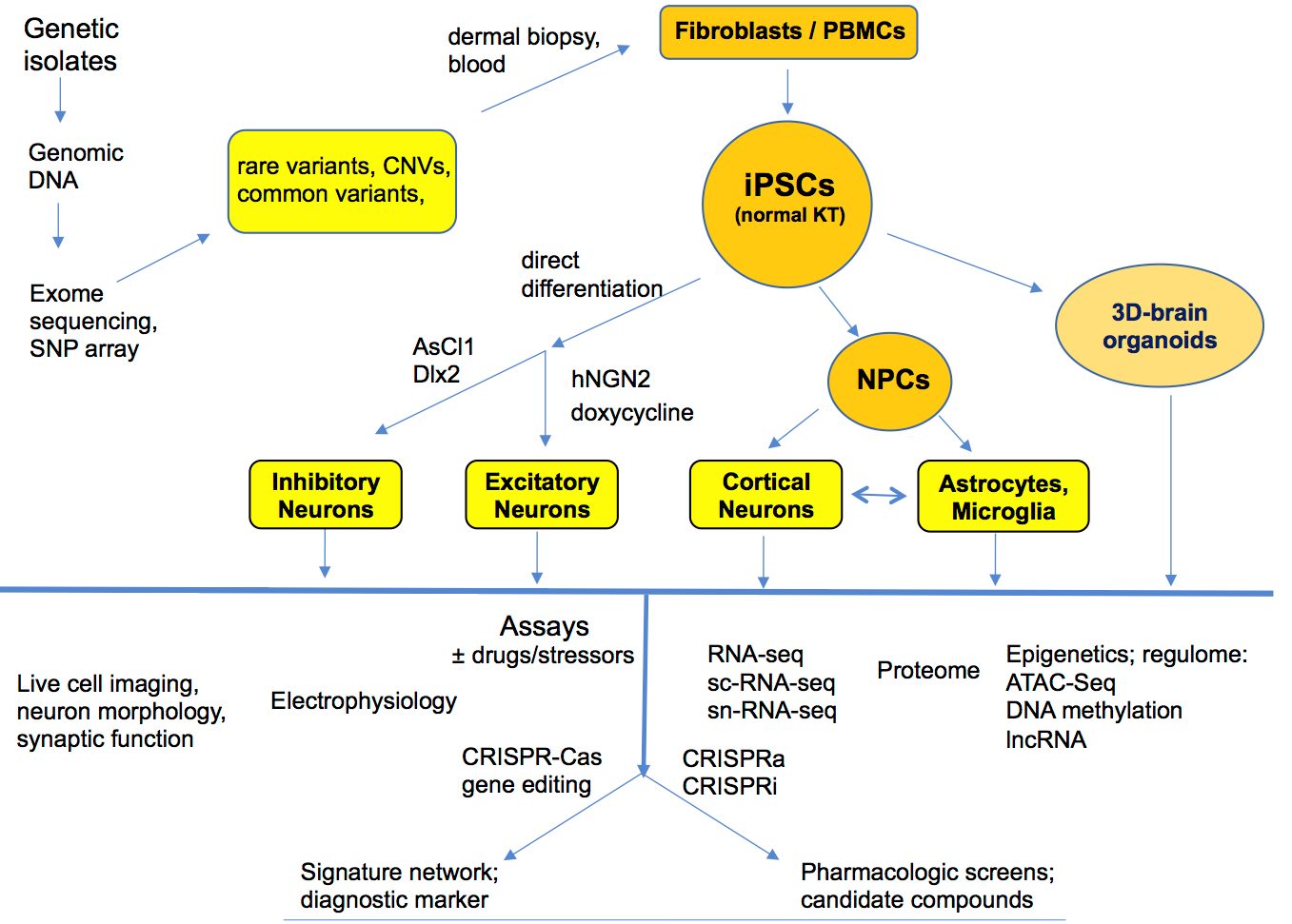

Supplement: Supplementary file 5 — Figure S2 [file 41398_2023_2641_MOESM5_ESM.png]
